# Does coping with pain help the elderly with cardiovascular disease? The association of sense of coherence, spiritual well-being and self-compassion with quality of life through the mediating role of pain self-efficacy

**DOI:** 10.1186/s12877-023-04083-x

**Published:** 2023-06-28

**Authors:** Nahid Salehi, Majid Yousefi Afrashteh, Mohammad Reza Majzoobi, Arash Ziapour, Parisa Janjani, Sahar Karami

**Affiliations:** 1 Cardiovascular Research Center, Health Institute, Imam-Ali hospital, Kermanshah University of Medical Sciences, Kermanshah, Iran; 2 Department of Psychology, Faculty of Humanities, University of Zanjan, Zanjan, Iran; 3 Developmental Psychology and Clinical Psychology of the Lifespan, , University of Siegen, Siegen, Germany

**Keywords:** Pain self-efficacy, Sense of coherence, Spiritual well-being, Self-Compassion, Quality of life, Aged, Cardiovascular Disease

## Abstract

**Background:**

Population ageing is considered one of the biggest challenges facing the world, and the status of the elderly in society and their quality of life (QOL) have proved to be a concern in professional and scientific research circles. As a result, the current study sought to investigate the role of pain self-efficacy (PSE) as a moderator in the relationship between sense of coherence (SOC), spiritual well-being, and self-compassion with QOL in Iranian elderly with cardiovascular disease (CVD).

**Method:**

This was a correlational study of the path analysis type. The statistical population included all elderly people with CVD who were at least 60 years of age in Kermanshah Province, Iran, in 2022, of whom 298 (181 men and 117 women) were selected using convenience sampling and according to the inclusion and exclusion criteria. The participants answered questionnaires from the World Health Organization on QOL, Paloutzian and Ellison’s spiritual well-being, Nicholas’s PSE, Antonovsky’s SOC, and Raes et al.’s self-compassion.

**Results:**

The results of path analysis demonstrated that the hypothesized model of this study has a good fit in the studied sample. There were significant paths between SOC (β = 0.39), spiritual well-being (β = 0.13) and self-compassion (β = 0.44) with PSE. Although there were significant paths between SOC (β = 0.16) and self-compassion (β = 0.31) with QOL, there was no significant path between spiritual well-being and QOL (β = 0.06). Besides, there was a significant path between PSE and QOL (β = 0.35). Finally, PSE was found to mediate the relationship of SOC, spiritual well-being and self-compassion with QOL.

**Conclusion:**

The results may provide psychotherapists and counselors working in this field of inquiry with advantageous information to choose or create a useful therapeutic method to work with the elderly with CVD. Meanwhile, other researchers are suggested to examine other variables which may serve a mediating role in the mentioned model.

## Background

Globally, the population is aging, and the elderly account for a larger share of the total population [1]. Meanwhile, age plays a critical role in the deterioration of cardiovascular function, thereby increasing the risk of cardiovascular disease (CVD) in the elderly [2]. Scientific evidence has revealed that the prevalence of CVD like atherosclerosis, stroke, and myocardial infarction increases with age in both men and women [3]. The American Heart Association reports that the incidence of CVD in US men and women is 40% in those aged 40–59 years, 75% in those aged 60–79 years, and 86% in those older than 80 years [4]. The burden of CVD in Iran is projected to increase sharply between 2005 and 2025, mainly due to the ageing of the population. Because of the increase in age, the disability-adjusted life years (DALYs) related to CVD in 2025 compared to 2005 will more than double [5]. Patients with CVD experience several physical symptoms, including fatigue, shortness of breath, or chest pain, which affect their physical, emotional, and social health and cause a significant disruption in their quality of life [6]. Moreover, dealing with a chronic disease such as CVD can change people’s lifestyles and have a significant effect on patients’ quality of life [7]. The results of a systematic review also confirm that cardiovascular changes in the elderly have adverse effects on their quality of life and longevity [8].

Quality of life (QOL) is defined, as the World Health Organization states, to be a broad concept that is affected in a complex way by physical health, mental state, level of independence, and social relationships [6, 9]. QOL can be considered one of the most important outcomes in the health care system, especially among patients with CVD [10]. Recently, a systematic review has shown that QOL in cardiac patients is moderate to poor [11]. In elderly patients with heart failure, low QOL has been reported [12]. Considering the impact of CVD on QOL, including increased mortality, problems returning to work, and disruption in performing daily tasks [13, 14], examining the determinants of QOL in people with CVD becomes important. The results of some studies have shown that QOL is related to basic variables such as depression [15, 16], anxiety [17, 18] and income [15] in patients with CVD. Recently, a great amount of evidence has emphasised the role of protective factors, suggesting that psychological resilience resources are associated with a lower risk of CVD and may promote healthy behaviours and cardiovascular health [19]. On top of all, the sense of coherence [20] has attracted considerable attention.

Sense of coherence (SOC) means a person’s effort to integrate his experience in the past, present, and future in order to adapt and improve himself [21]. SOC enables people to identify and use the resources available to them. The more a person can understand and manage the significance of a stressful situation or disease, the more their potential to successfully cope with it increases [20]. Indeed, SOC is an important concept for understanding individual differences in coping with stress [22]. Previous studies have also reported an association between a strong SOC and a physically active lifestyle in the general population [23–25] and among post-myocardial infarction patients [26, 27]. Some studies have also reported an association between better QOL and a stronger SOC among patients with chronic diseases such as heart disease, and there is evidence to support a positive relationship between SOC and QOL [28–31]. The results of other studies also emphasise the importance of the SOC as a strong predictor of QOL in the elderly [32, 33]. Another variable related to QOL appears to be spiritual well-being.

Spiritual well-being is a concept for which there is no consensus on the specific definition, but recently a conceptual framework of spiritual needs was designed by Büssing et al. [34], which includes four basic needs: religious needs, the need for inner peace, the need for existence, and the need to actively forgive. In fact, religious practises and spiritual beliefs somehow affect people’s coping and adaptation mechanisms in dealing with various chronic diseases [35–37], and this issue can affect people’s QOL. The results of some studies have also shown that there is a relationship between spiritual well-being and QOL [38, 39]. The results of a systematic review also confirm that there is an inherent connection between spiritual well-being and QOL in cardiovascular patients [6], Another variable that can be related to the QOL of patients with CVD is self-compassion.

From the point of view of Neff [40], a pioneer of experimental work, “self-compassion” is having a non-judgmental attitude towards one’s shortcomings, weaknesses, failures, pain, and suffering, not avoiding them, and the desire to reduce pain and suffering and self-heal with kindness. Although self-compassion is associated with psychological outcomes, there are a limited number of studies examining the relationship between self-compassion and QOL in patients whose physical health is threatened [41], and to the best of our knowledge, only one study showed that higher self-compassion is associated with fewer clinical symptoms of CVD [42]. In order to understand and clarify the relationship between the structures of SOC, spiritual well-being, and self-compassion with QOL, it seems that one of the most important and effective factors is pain self-efficacy.

Self-efficacy is one of the main constructs of social cognition theory, which means how confident the patient is in performing their abilities [43]. Pain self-efficacy (PSE) is one of the psychological variables of pain, which shows a person’s confidence in his ability to maintain function despite the presence of pain [44]. In fact, self-efficacy increases a person’s capacity to cope with pain [45]. Self-efficacy as an effective factor on QOL emphasises people’s understanding of their skills [46], and it can affect QOL in patients with chronic diseases such as diabetes or CVD due to the role it plays in managing healthy behaviour and adapting to a healthy lifestyle [47, 48]. Accordingly, the results of a study in Palestine also confirm that lower levels of cardiac self-efficacy predict a lower QOL in patients with coronary heart disease [49]. Other results confirm the relationship between lower levels of cardiac self-efficacy [50] and lower quality of life in patients with coronary heart disease.

Among the studies with path analysis methods that have been conducted in recent years in the field of identifying mediating variables in the relationship between spiritual well-being, SOC, and self-compassion with QOL, we can mention the research conducted by Sharifinia et al., the results of which revealed that the relationship between spiritual well-being and QOL in patients with cancer can be mediated by hope.

**Fig. 1:**
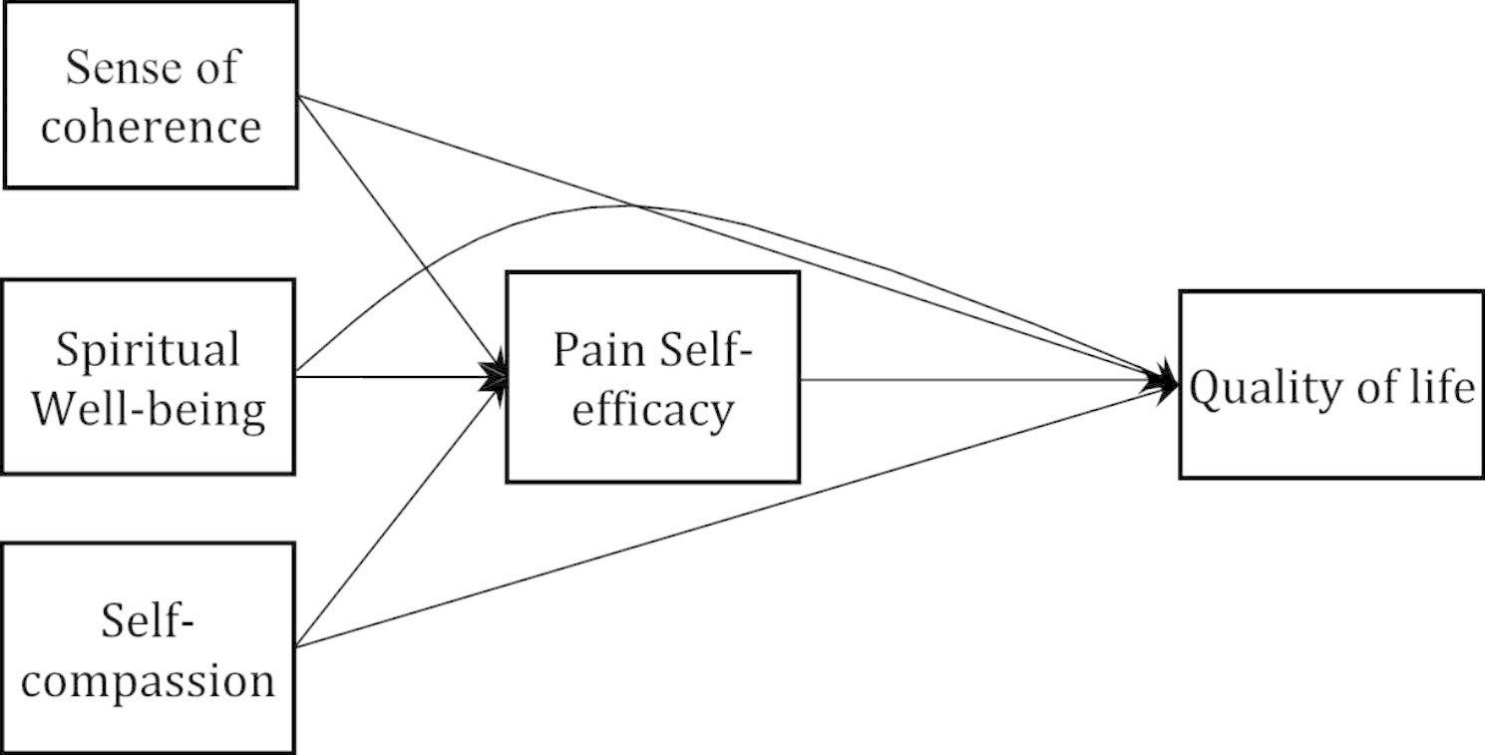
Hypothesized model for the relationship between SOC, Spiritual Well-being and Self-compassion with QOL through Pain Self-efficacy

Therefore, because the issue of QOL is discussed as a significant health consequence in the management of any disease, including CVD, and because the prevalence of CVD, especially in the elderly, imposes a major economic burden on the health care infrastructure, the study of QOL in the elderly with CVD appears to be a priority. Hence, the importance of identifying direct and indirect predictors of QOL in the elderly community with heart disease is obvious. As the current study examines both the elderly and people with CVD, the value of that seems to be doubly important. Providing models in which the predictor variables of the quality of life along with the mediator variables through which the predictor variables are associated to the quality of life in the elderly with CVD, can provide highly advantageous information for psychologists and counselors working in this field of inquiry. As such, they can use those type of therapeutic protocols that focus on variables like sense of coherence, spiritual well-being, and self-compassion, and by improving these variables, they can help the elderly with CVD to improve their quality of life. Therefore, conducting fundamental studies like the current one that provide primary models of quality of life appears highly necessary in terms of their scientific, therapeutic and social importance. Consequently, the present study was conducted with the aim of investigating the mediating role of PSE in the relationship between SOC, spiritual well-being, and self-compassion with QOL in elderly patients with CVD. The hypotheses of this study were that (1) SOC is related to QOL through PSE, (2) spiritual well-being is related to QOL through PSE, and (3) self-compassion is related to QOL through PSE. The hypothesised model of the study can be seen in Fig. 1.

## Method

### Study design and participants

The method of the current study was correlational path analysis. The study population included all the elderly with CVD living in Kermanshah city in 2022, of whom 298 (181 men and 117 women) elderly with CVD referring to Imam Ali Hospital with a stable condition were selected using convenience sampling. Inclusion criteria included (1) age 60 and above, (2) informed consent, and (3) a diagnosis of CVD. Exclusion criteria include (1) open heart surgery (CABG), (2) heart failure, (3) history of myocardial infarction in the past 6 months, (4) history of drug abuse, (5) cancer diagnosis, (6) presence of congenital heart disease, (7) experience of bereavement of loved ones in the past 6 months, (8) Corona disease in the last two months, (9) history of previous hospitalisation due to heart disease, and (10) the presence of any severe psychological disorder based on the medical records of the individuals (in order to check this criterion, the participants were asked if there was a history of diseases such as Alzheimer’s disease or dementia in their records.

### Sampling and sample size

Regarding the sample size, it is necessary to explain that in the analysis of Stevens [51], 15 cases for each predictor variable in the multiple regression analysis with the standard least squares method are considered a rule of thumb. Accordingly, it can be stated that because path analysis is completely related to multivariate regression in some aspects, 15 items for each measured variable in path analysis is not unreasonable. Loehlin [52] states that for models with two or four factors, the researcher should plan on collecting at least 100 cases or more, about 200. Therefore, taking into account 15 participants for each component and considering that the current research included 18 components, the minimum number of samples required to conduct the study is 270. By taking into account factors such as incomplete or distorted questionnaires, 10% was considered a dropout, and the questionnaire was given to 300 people.

### Measures

Reagrding the questionnairs applie in the current study, it is worthy of mention that although there may found a long form for all the questionnairs mentioned below, the main critrion for choosing questionnair in the current study was the number of items exsist in each questionnair. As we needed participants to fill out five questionnair, the application of long-form questionnaires could have caused the elderly to refuse completing the questionnaires. To the best of our knowledge based on previous experiences, the longer questionnaires are used, the more participants drop out.

#### Demographics collection sheet

Using this sheet, we asked participants to provide their demographic information, including age, level of education, marital status, occupation, and income.

#### Quality of life questionnaire (short form)

The 12-item version of the QOL questionnaire, designed by Ware et al., has eight subscales (four physical and four psychological) [53]. The physical component includes the overall perception of one’s health, physical functioning, physical health, and physical pain. The psychological component includes emotional problems, social functioning, vitality and vital energy, and mental health. Due to the small number of items, the individual’s overall score is often used. To fill out the questionnaire, participants rate the items on a 5-point Likert scale from 1 to 5. The minimum and maximum scores are 12 and 60, respectively, with a higher score indicating a better quality of life. Using Cronbach’s alpha coefficient, Ware et al. showed that the reliability of physical and psychological components was 0.89 and 0.76, respectively [53]. In Iran, Montazeri et al. reported the reliability of physical and psychological components based on Cronbach’s alpha coefficient to be 0.73 and 0.72, respectively [54]. In the current study, the Cronbach’s alpha coefficients for the physical and psychological components were 0.77 and 0.74, respectively.

#### Sense of coherence questionnaire (short form)

The SOC questionnaire, prepared by Antonovsky [55], has 29 items. In this study, the short form of the questionnaire, with 13 questions, was used. Participants rate the items on a 7-point Likert scale from 1 to 7. A score between 13 and 26 means a low SOC; a score between 26 and 52 means a medium SOC; and a score above 52 means a high SOC. In a systematic review of 458 scientific articles and 13 doctoral theses between 1992 and 2003, Eriksson et al. concluded that the SOC questionnaire (29 questions and 13 questions) is reliable and valid [56]. In Iran, the 13-question form has been validated by Mahammadzadeh et al., and its Cronbach’s alpha was 0.77. In the current study, the Cronbach’s alpha coefficient for the SOC questionnaire was 0.79 [57].

#### Spiritual well-being scale (SWBS)

Paloutzian and Ellison created this questionnaire, which consists of 20 items, 10 of which assess religious health and the remaining 10 assess existential health.Participants’ responses to the items on a 6-point Likert scale ranged from strongly disagreeing (1 point) to strongly agreeing (6 points). The minimum and maximum scores are 20 and 120, respectively. Spiritual health is classified into three levels: low (40–20), medium (99–41), and high (120–100). Paloutzian and Ellison considered this questionnaire valid and reported Cronbach’s alpha coefficients for religious and existential health and the total score as 0.91, 0.91, and 0.93, respectively [58]. In Iran, Allahbakhshian et al. reported a Cronbach’s alpha of 0.82 for the total score of this questionnaire [59]. In the current study, Cronbach’s alpha coefficients for religious health, existential health, and spiritual well-being were 0.81, 0.76, and 0.80, respectively.

#### Short form of self-compassion questionnaire (SCS-SF)

The SCS-SF, developed and validated by Raes et al., has 12 items and three subscales: self-kindness-self-judgement, common humanity-isolation, and mindfulness-overall. The items have been scored on a 5-point Likert scale, from never (1) to always (5) [60]. The minimum and maximum scores are 12 and 60, respectively, with a higher score indicating higher self-compassion. Raes et al. [60] calculated the internal reliability of the SCS-SF using Cronbach’s alpha coefficient to be 0.86 and stated that it has an adequate correlation with the long form of this questionnaire. In Iran, Khanjani et al. reported Cronbach’s alpha coefficient for the subscales of self-kindness and self-judgement, common humanity and isolation, and mindfulness and overidentification, as well as the total score, to be 0.68, 0.71, 0.86, and 0.79, respectively [61]. Khanjani et al. also reported test-retest reliability with a one-week interval to be 0.90. In the current study, Cronbach’s alpha coefficient for the total score was 0.84.

#### The pain self-efficacy questionnaire

This questionnaire, compiled by Nicholas based on Bandura’s theory, evaluates the patient’s belief in his ability to perform various activities despite the presence of pain. This questionnaire evaluates the efficiency and sufficiency of a person’s life with pain. Participants responded to this single-factor questionnaire on a 6-point Likert scale, ranging from zero (I am not sure at all) to six (I am completely sure), with scores ranging from 1 to 61. A higher score indicates a strong belief in doing daily activities despite the presence of pain [44]. Asghari & Nicholas considered the validity and reliability of the Persian version of the PSE questionnaire using confirmatory factor analysis in a sample of 348 patients with chronic pain; the results showed that the single-factor version of the PSE questionnaire has a good fit in the studied population [62]. Asghari & Nicholas also reported the Cronbach’s alpha of the PSE questionnaire to be 0.92 [62]. Abedi Ghelich Gheshlaghi et al. reported the reliability of the questionnaire using three methods of test-retest with a three-day interval, Cronbach’s alpha coefficient, and halving to be 0.81, 0.78, and 0.77, respectively, which indicates the satisfactory reliability of the questionnaire [63]. Cronbach’s alpha was calculated to be 0.78 in the current study.

### Data collection procedure

After obtaining permission from the Ethics Committee of Kermanshah University of Medical Sciences and obtaining other necessary permissions to conduct the research from competent authorities, the preliminary stage of the research was carried out. As several questionnaires were available for measuring the variables of this research, the existing questionnaires were considered, and among them the questionnaires whose questions and subscales were closer to the objectives of the study were selected. Meanwhile, taking into account that patients with CVD, especially the elderly, are in special physical and mental conditions due to the burden of the disease, and in order to increase the accuracy of the study and receive more correct and accurate answers, we tried to use the shortest form of the questionnaires, if possible, to conduct this study. The questionnaires having been designed in the Digit system (an online questionnaire on the web), a trained interviewer invited eligible patients to participate in the research and complete the questionnaire by referring to the inpatient departments. After justifying the research to the participants, explaining the objectives, and obtaining the consent form, as well as reassuring them about the confidentiality of the answers and the need not to mention names, it was emphasised that the participants complete and accurately fill out the questionnaires.The participants were requested to ask the researcher for further explanation if they encountered a problem such as ambiguity in the questions in the process of completing the questionnaire. Moreover, patients were asked to answer the questions when they had the right conditions. After collecting information and removing two distorted questionnaires (a software problem in data recording), Therefore, the data of 298 participants was analysed using Pearson’s correlation coefficient and path analysis in SPSS 26 and LISREL 10.2 software. It needs to be mentioned that the sex and age of the participants were considered to be control variables.

## Results

Table 1 shows the demographic characteristics of the participants.

**Table 1:** Demographic characteristics of the participants in the study

Variable	frequency	Percentage
Gender		
Man	181	61
Woman	117	39
Habitation		
Urban	242	81
Rural	56	19
Job Status		
Unemployment	59	19.8
Employed	32	10.7
Self-employment	93	31.2
Housekeeper	113	37.9
Other	1	0.3
Marital Status		
Married	278	93.3
Single	1	0.3
Widow	19	6.4
Education level		
Iliterate	193	64.8
Elementary	62	20.8
First secondary	21	7
Senior secondary	11	3.7
University	11	3.7
Income		
< 2	2	0.7
2–4	20	6.7
4–6	125	41.9
> 6	41	13.8
5	110	36.9

Table 2 includes descriptive data, such as mean and standard deviation, as well as a correlation matrix for the relationship between variables.

**Table 2:** Mean, standard deviation and correlation matrix using Pearson’s correlation coefficients

Variable	M	SD	1	2	3	4
1. SOC	49.80	4.53	-			
2. Spiritual Well-being	56.34	5.94	0.20**	-		
3. Self-compassion	35.71	4.34	0.58**	0.13*	-	
4. PSE	30.68	7.19	0.63**	0.14*	0.66**	-
5. Quality of life	86.19	10.02	0.56**	0.14**	0.63**	0.65**

*=p < 0.01 **=p < 0.05

According to Table 2, all Pearson’s correlation coefficients were significant for the variable relationship. Considering control variables, sex (r = 0.15, p < 0.007) was significantly correlated with QOL, while age (r = 0.03, p < 0.62) was not significantly correlated with QOL. Therefore, in the subsequent path analysis and mediation tests, sex was included as a control variable. The results of Pearson’s correlation coefficient provide preliminary support for the hypotheses. After considering sex as a control variable in the path analysis, the model fit indices reported in Table 3, the hypothesised model fit was good. According to Table 3, all indices are in good condition. Regarding, it is worth mentioning that the skewness index for the research variables was between − 1 and + 1, which indicates that the distribution of variables is normal. Therefore, there was no limitation to the use of Pearson’s correlation coefficient and path analysis.Path analysis with ordinal data was conducted using the diagonally weighted least squares method (WLSMV). We examined the proposed model using all the data obtained from the original questionnaire,and the model fit indices including Chi square statistics, Chi square/df,Comparative Fit Index (CFI), Root Mean Square Error of Approximation (RMSEA), Tucker-Lewis Index [TLI, also known as the Non-normed fit index (NNFI)], Goodness of Fit Index (AGFI) and Adjusted Goodness of Fit Index (AGFI) were considered. When the RMSEA ≤ 0.05, CFI and TLI ≥ 0.95, and WRMR < 0.90 the model was judged as a good fit.

**Table 3:** Indexes of model fit

Index	Value	Criteria value	Situation
X2	0.63	-	-
df	1	-	-
P	0.42	> 0.05	good
X2/df	0.63	< 3	good
RMSEA	0.01	< 0.05	good
GFI	0.96	> 0.90	good
AGFI	0.98	> 0.90	good
TLI	0.95	> 0.90	good
NFI	0.98	> 0.90	good
CFI	0.98	> 0.90	good

The results of path analysis to determine the relationships between variables and investigate the direct, indirect, and total effects are reported in Table 4.

**Table 4:** Path coefficients for direct, indirect and total SOC, Spritual well-being, self compasion and PSE with QOL

Pathways	Standard Estimate	t-value	P-value
Direct			
SOC → QOL	0.16	2.21	P < 0.05
Spiritual Well-being → QOL	0.06	1.21	P = 0.12
Self-compassion → QOL	0.31	5.44	P < 0.001
PSE → QOL	0.35	5.98	P < 0.001
SOC → PSE	0.39	7.84	P < 0.001
Spiritual Well-being → PSE	0.13	2.14	P < 0.05
Self-compassion → PSE	0.44	9.05	P < 0.001
Indirect			
SOC → PSE → QOL	0.14	4.75	P < 0.001
Spiritual Well-being → PSE → QOL	0.05	2.06	P < 0.05
Self-compassion → PSE → QOL	0.16	4.99	P < 0.001
Total			
SOC→ QOL	0.30	5.61	P < 0.001
Spiritual Well-being → QOL	0.11	2.68	P < 0.001
Self-compassion → QOL	0.46	8.76	

**Fig. 2:**
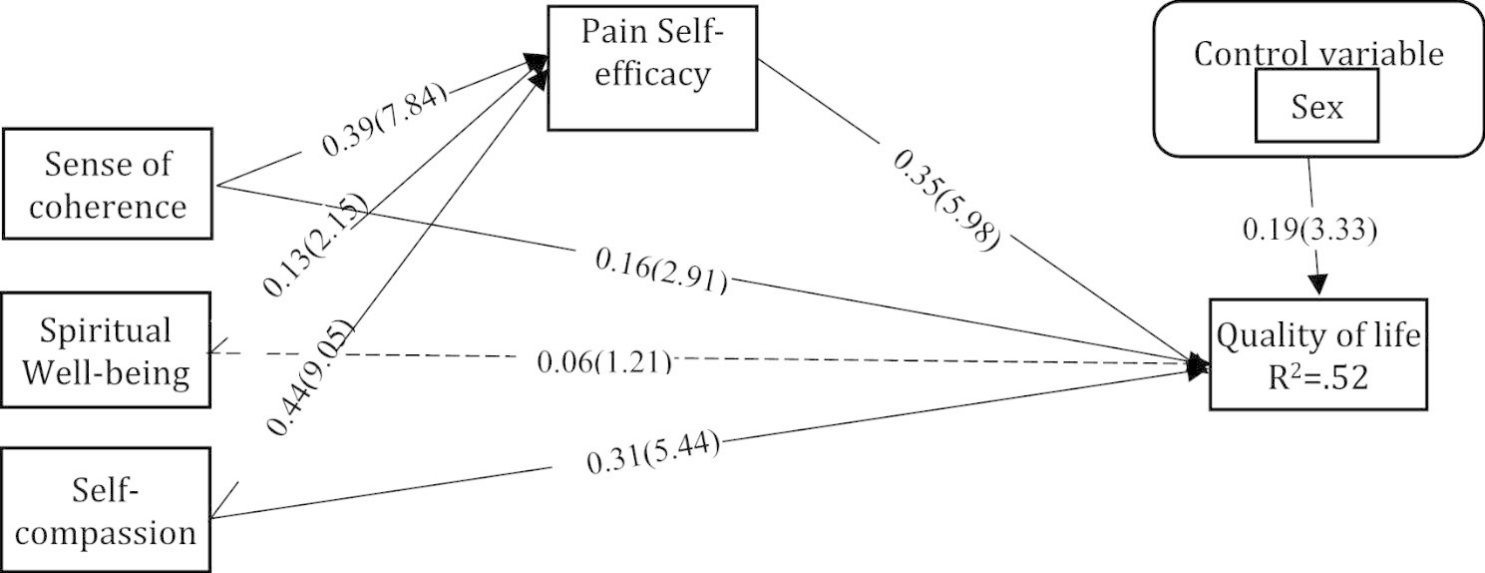
Path diagram for the relationship between SOC, Spiritual Well-being and Self-compassion with QOL through Pain Self-efficacy

Table 4 shows the direct, indirect, and total effects of the relationships among the variables in the model. According to the results of this table, only the direct link between spiritual well-being and QOL is significant. The rest of the path is significant. Thus, PSE mediates the relationship between SOC, spiritual well-being, and self-compassion with QOL. Figure 2 shows the final model of the study.

## Discussion

The present study was conducted with the aim of investigating the relationship between SOC, spiritual well-being, and self-compassion with QOL through the mediating role of PSE in the elderly with CVD in Kermanshah. The results of the path analysis showed that the hypothesised model of this study has a good fit in the studied sample. According to the findings, in addition to the direct effect of SOC and self-compassion on QOL, their indirect effect and the indirect effect of spiritual well-being on QOL were confirmed via the mediating role of PSE.Thus, all the hypotheses of this research were confirmed.

The first hypothesis of this study, that SOC is related to QOL through PSE, was confirmed. In other words, people with a higher SOC have a higher PSE, which in turn leads to a better QOL. Although no study has investigated this path, in terms of the relationships that make up this path, the findings of this study are in line with previous studies in the field of the direct relationship between QOL and SOC in patients with chronic diseases such as CVD [29, 31] and the elderly [32, 33]. Considering that the SOC is an important concept for understanding individual differences in dealing with stress [22], in explaining this finding, it can be said that when a person’s point of view is that he cannot be affected by external and internal problems, it is obvious that he has high self-confidence and can overcome problems, pressures, and all kinds of stress. In fact, being away from and able to overcome stress is an important factor for increasing QOL. Thus, a person who has a strong SOC when facing a stressful factor will have the ability to understand and manage the situation using available resources, which will lead to the development and maintenance of his health. This increases satisfaction and QOL by reducing pressure and tension [64]. So, in general, it can be said that the feeling of high coherence, increasing adaptability and control, and overcoming stress can play a role in increasing people’s QOL. Further strengthening of this sense affects not only health but also QOL and improves it [56, 65]. On the other hand, due to the fact that PSE also refers to the level of a person’s confidence in his ability to maintain functioning despite the presence of pain [44], the SOC can indirectly increase that through the ability to understand and manage the situation in stressful events, and with the role it plays in managing healthy behaviour and adapting to a healthy lifestyle in patients with chronic diseases such as diabetes or heart disease, it can affect QOL [47, 48].

The second hypothesis of this study, that spiritual well-being is related to QOL through PSE, was confirmed. In other words, the elderly with CVD who report higher spiritual well-being have higher PSE, which in turn is directly related to better QOL. The results of the present study are in line with the findings of previous studies [57, 66], and the results of their study also confirm that spiritual well-being predicts QOL, and various mechanisms confirm this relationship. The results of a systematic review also confirm that there is an inherent relationship between spiritual well-being and QOL in patients with CVD [6]. Due to its chronicity and debilitating nature, CVD negatively affects the patient’s life satisfaction and imposes a lot of psychological pressure on the patient [67]. The chronic nature of this disease affects all aspects of the patient’s life, including personal, social, family, and work, and affects people’s QOL. Obviously, such conditions in the lives of the elderly bring about more limitations and negative consequences. Spirituality increases the ability to cope with illness and the speed of recovery [68]. It seems that due to having meaning in life, hoping for God’s help in critical situations, benefiting from social and spiritual support, and feeling like they belong to a superior source, religious people undergo less pressure in the face of harmful events such as contracting a disease [68]. In fact, religion creates a positive attitude about the world and helps a person face unfortunate life events such as loss or illness. On the other hand, religion gives meaning and purpose to one’s life, and having meaning and purpose in life indicates mental health and increases one’s ability to do things [69]. Accordingly, some researchers are of the opinion that in the treatment of the disease, it is necessary to consider the patient as a whole and in all aspects, and if only his physical condition is considered, the healing process will be disturbed [70]. In fact, religion and spirituality are important sources of strength and support throughout life and help to get out of critical and stressful situations, especially in the elderly population. Spiritual affairs provide a source of support for the elderly that can help them deal with challenging life factors. Therefore, the elderly who have stronger religious beliefs, by emphasising their source of support, consider themselves more capable of solving life’s problems, do not give up early, and have better endurance. This issue can improve the QOL of patients.

The third hypothesis of this study, that self-compassion is related to QOL through PSE, was confirmed. In other words, self-compassion, both directly and indirectly through PSE, predicts the quality of life of the elderly with CVD. Self-compassion is a positive psychological construct characterised by the expansion of self-compassion, often during periods of suffering. While self-compassion has been linked to psychological outcomes, research linking self-compassion to QOL in patients whose physical health is threatened is limited [41]. Having a chronic disease can cause changes in patients’ lives. Self-compassionate people prepare themselves for life changes and correct their harmful and undesirable behaviour patterns. Therefore, self-compassion can be considered in different ways as an emotion regulation strategy in which the experience of annoying and undesirable emotions is not prevented but rather accepted in a kind way. Therefore, negative emotions change to positive ones and the person finds new ways to face the disease, and this can affect QOL.

### Implications of the study for treatment

By quickly reviewing the interpretations provided for the hypotheses of this study, one can understand the significance of a basic issue common to all of people with CVD that has received little attention, and this issue is nothing but PSE, which is one of the psychological variables of pain that shows a person’s confidence in his ability to maintain performance despite the presence of pain [44] and increases a person’s capacity to deal with pain [45]. Because of the complex nature of chronic pain, the current approach to pain treatment now outweighs the physical and pharmacological approaches, and the significance of psychological factors in chronic pain management has received attention. As self-efficacy training in general and in any field provides belief in ability and an optimistic outlook on life, it is one of the constructive elements of action that restores belief and gives people the courage to do work, and through encouragement, a person becomes aware of his values and recognises his strengths and assets. As a consequence, self-efficacy training enhances empathy, and as a result, people’s QOL improves.

### Limitations and suggestions

The cross-sectional design of this research is one of its drawbacks, and causal findings from this sort of study are not acceptable. Given the limits of the current investigation, it is proposed that future studies analyse the association shown in this study using highly accurate techniques in a setting where disturbing factors are controlled. Future research might also employ longitudinal designs and cohort studies to establish a causal link between these psychological dimensions.

## Conclusion

The results of this study emphasise the importance of the role of PSE in increasing the quality of life of the elderly with CVD. Based on this, the present study can have a very clear message for specialists and therapists working in the field of the elderly that is the use of interventional and therapeutic methods in the field of increasing PSE, which can increase QOL of the elderly suffering from CVD. Although the path model presented in the current study provide no causal relationship between the predictor variables with the mediating and criterion variable, it can help the psychotherapists working with the elderly to have clear idea about the interrelations between the mentioned varibales. Therefore, having read the current study, psychotherapists would probably realize that by using theraputic protocols availabe for enhancing the sense of coherence, spiritual well-being and self-compassion, they can help the older people with CVD to cope better with pain, which, in turn, may lead the elderly to have better quality of life. Besides, they can directly take advantages of protocols made for inhancing PSE in order to improve QOL in the elderly. To put in a nutshell, the fundamental studies like the current study can provide the relationship between the crutial variables and the provides relationships can help researchers and thertapists to either make some theraputice protocol or apply the available theraputice protocol to enhance those crutial variables in the elderly.

## Data Availability

The datasets used in the study are available from the corresponding author on reasonable request.
